# Metabolism and the Evolution of Social Behavior

**DOI:** 10.1093/molbev/msx174

**Published:** 2017-06-08

**Authors:** Kerry E. Boyle, Hilary T. Monaco, Maxime Deforet, Jinyuan Yan, Zhe Wang, Kyu Rhee, Joao B. Xavier

**Affiliations:** 1 Program in Computational and Systems Biology, Memorial Sloan-Kettering Cancer Center, New York, NY; 2 Program in Immunology and Microbial Pathogenesis, Weill Cornell Graduate School of Medical Sciences, New York, NY; 3 Tri-Institutional Training Program in Computational Biology and Medicine, New York, NY; 4 Department of Medicine, Weill Cornell Medical College, New York, NY

**Keywords:** metabolomics, bacteria, social evolution theory, genome sequencing, experimental evolution, swarming

## Abstract

How does metabolism influence social behavior? This fundamental question at the interface of molecular biology and social evolution is hard to address with experiments in animals, and therefore, we turned to a simple microbial system: swarming in the bacterium *Pseudomonas aeruginosa*. Using genetic engineering, we excised a locus encoding a key metabolic regulator and disrupted *P. aeruginosa*’s metabolic prudence, the regulatory mechanism that controls expression of swarming public goods and protects this social behavior from exploitation by cheaters. Then, using experimental evolution, we followed the joint evolution of the genome, the metabolome and the social behavior as swarming re-evolved. New variants emerged spontaneously with mutations that reorganized the metabolome and compensated in distinct ways for the disrupted metabolic prudence. These experiments with a unicellular organism provide a detailed view of how metabolism—currency of all physiological processes—can determine the costs and benefits of a social behavior and ultimately influence how an organism behaves towards other organisms of the same species.

## Introduction

Metabolism influences the way individuals behave toward others. In all species, from bacteria to animals including humans, social behavior appears to be a function of metabolic state (Robinson et al. 2008; Biro and Stamps 2010; Boyle et al. 2015; Sih et al. 2015). Organisms tend to be more cooperative when their metabolic reserves are full. Vampire bats share blood with their starving roost-mates, but they share more when they have fed well (Carter and Wilkinson 2013); bacteria send and receive more chemical quorum-sensing signals to one another when they have more intracellular metabolites (Xavier and Bassler 2005); and judges give more favorable parole decisions when they resume their work after a meal (Danziger et al. 2011). Why is it that—across the tree of life—metabolism seems to condition social behavior?

A social behavior is any behavior that involves interactions among members of the same species and influences their reproduction and survival (Robinson et al. 2008). For a social behavior to evolve by natural selection it must have a low cost-to-benefit ratio, where the fitness cost on the actor’s genotype takes into account the indirect fitness benefit given to the recipient (Hamilton 1964; Nowak 2006; Gardner et al. 2011). For the same benefit to the recipient, natural selection should reduce the cost on the actor. Since metabolism is the currency of all physiological processes that support life (Smith and Morowitz 2004) and a major determinant of behavioral cost (Biro and Stamps 2010), natural selection should favor a regulation of social behavior that reduces metabolic burden on the actor.

Here, we investigated this problem experimentally in a microbial model of social behavior: swarming motility in the bacterium *Pseudomonas aeruginosa*. In order to swarm, *P. aeruginosa* must synthesize and secrete massive amounts of rhamnolipid biosurfactants that make a thin lubricating film on which billions of bacterial cells rotating their flagella can move collectively (Deziel et al. 2003; Caiazza et al. 2005). Swarming helps the colony forage more nutrients; in the laboratory, colonies of wild-type bacteria swarm to become 7 times more populous than colonies of nonswarming mutants lacking rhamnolipid synthesis such as the genetically engineered Δ*rhlA* mutant (Xavier et al. 2011). Rhamnolipid secretion could be a significant metabolic burden because bacteria can produce >20% of their own biomass in these surfactants (Guerra-Santos et al. 1984) that once secreted become a public good. Wild-type *P. aeruginosa* avoid wasting valuable metabolic resources, and potential exploitation by Δ*rhlA* cheaters, by expressing *rhlA* only when the colony is large enough and individuals have more carbon than they can possibly use for growth. This regulatory strategy is called metabolic prudence (Xavier et al. 2011) and it stabilizes swarming against cheating because it enables bacteria to delay the production of the carbon-rich rhamnolipids to times when their growth is limited by lack of another essential nutrient, such as nitrogen or iron (Mellbye and Schuster 2014). Regulation of *rhlA* integrates quorum-sensing signals with information on the metabolic state of the cell to turn on expression only when population density is high and there is an excess of carbon, and expression shuts off immediately when carbon is low (Boyle et al. 2015). Metabolic prudence reduces the cost-to-benefit ratio of swarming, ensuring that this social behavior is protected from exploitation by nonrhamnolipid cheaters across a wide range of conditions (de Vargas Roditi et al. 2013).

**Fig. 1.**
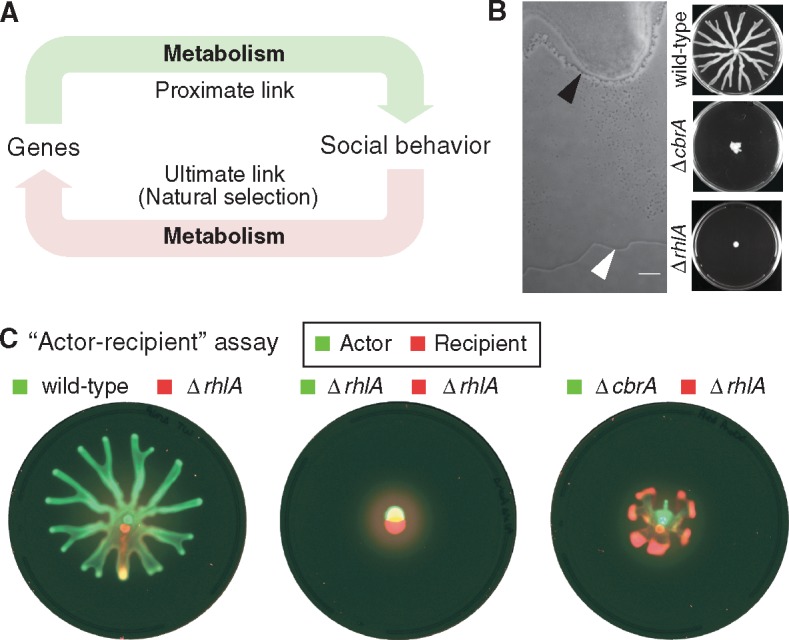
Swarming in *Pseudomonas aeruginosa*: A model of metabolism and social behavior. (*A*) Metabolism is the currency of all cellular processes, but its role in the feedback between genes and social behavior remains poorly studied. (*B*) Swarming in *P. aeruginosa* requires the production of rhamnolipid surfactants. Left: detail of the leading edge in a swarming colony with black arrow head indicating the edge of the colony and the white arrow head indicating the edge of the rhamnolipids produced by the bacteria—the public good required for swarming (scale 100 µm, see supplementary video S1, Supplementary Material online). Right: swarming in wild-type, lack of swarming in the strain Δ*cbrA* which we engineered to perturb the prudent regulation of rhamnolipid synthesis and the Δ*rhlA* strain which lacks rhamnolipid synthesis entirely (9 cm petri dishes shown). (*C*) Social behavior assays with the Δ*rhlA* nonswarmer reveal that Δ*cbrA* strain can produce biosurfactants that help Δ*rhlA* swarm.

We used the swarming system to conduct a perturbation experiment on organismal social behavior: We disrupted metabolic prudence by genetically engineering a deletion in the key metabolic-regulatory locus, *cbrA*. Then, we used metabolomics and genome sequencing to follow the concerted evolution of the metabolome, the genome and the social behavior as spontaneous mutants emerged to take over the population. Our experiments—to the best of our knowledge, the first to apply metabolomics to social evolution—revealed that in the absence of proper metabolic regulation the recovery of the original metabolomic state does not necessarily guarantee the best social behavior. More generally, experimental evolution in microbes provides a unique view on how metabolism plays a key role in the intricate feedback between genes and social behavior (fig. 1*A*).

## Results

### Excision at *cbrA* Locus Disrupts Metabolic Prudence and Produces a Synthetic Altruist

Swarming is a social behavior that relies on the collective secretion of rhamnolipid surfactants (fig. 1*B*, left panel and supplementary video S1, Supplementary Material online); metabolic prudence regulates expression of the *rhlA* gene for rhamnolipid synthesis, ensuring that *P. aeruginosa* produces these surfactant public goods only when it has carbon available in excess relative to other nutrients such as nitrogen. Intracellular balance of carbon and nitrogen metabolism is in part ensured by the two-component system CbrA/CbrB (Li and Lu 2007; Zhang et al. 2015). Previous work had shown that disrupting *cbrA* affects swarming, slows bacterial growth in nonpreferred carbon and nitrogen sources and disrupts metabolism (Nishijyo et al. 2001; Li and Lu 2007; Yeung et al. 2011; Zhang et al. 2015). We set out to determine how disruption of *cbrA* would affect metabolic prudence.

We engineered a deletion at the *cbrA* locus in *P. aeruginosa* PA14. The strain we used harbored the reporter fusion P_*rhlAB*_-*gfp* so that we could quickly measure the effect on *rhlA* expression by measuring green fluorescence (supplementary fig. S1, Supplementary Material online). The phenotype of the engineered strain Δ*cbrA*:P_*rhlAB*_-*gfp* (from hereon called simply Δ*cbrA*) was consistent with the phenotypes previously reported: the strain swarmed only marginally better than a nonswarming Δ*rhlA* strain (fig. 1*B*) and grew slower than the wild-type in liquid media with casamino acids as the sole carbon and nitrogen source (supplementary fig. S2A, Supplementary Material online). The lack of swarming was likely caused by the slower growth in casamino acids which are also the sole carbon and nitrogen source in swarming assays; the bacteria still had the flagella machinery necessary to swim (supplementary fig. S3, Supplementary Material online) and expressed rhamnolipid genes at even higher levels than the wild-type PA14 (supplementary fig. S2B, Supplementary Material online). In fact, the Δ*cbrA* secreted such copious amounts of rhamnolipids that it could help the nonproducer Δ*rhlA* strain swarm, despite being unable to swarm on its own (fig. 1*C*).

To determine the costs and benefits of the apparently altruistic behavior of Δ*cbrA*, we developed a new quantitative “actor-recipient” assay. We mixed our green-labeled Δ*cbrA* strain (the actor) with a red-labeled Δ*rhlA* (the recipient). The different fluorescent labels allowed us to measure the numbers of each strain before and after swarming competition and quantify the net fitness benefit to the actor as the change in frequency of Δ*cbrA*. We then determined the benefit to the recipient by the ratio of the final number of the red-labeled Δ*rhlA* cells to the final number of red-labeled Δ*rhlA* cells achieved in a control experiment where we mixed the red-labeled Δ*rhlA* with a neutral competitor, a green-labeled version of Δ*rhlA*. According to this “actor-recipient” assay, the actor Δ*cbrA* gave a large benefit to the Δ*rhlA* recipient by helping it swarm, but the Δ*cbrA* itself had a net negative benefit, i.e., it incurred a fitness cost (supplementary fig. S4, Supplementary Material online). Therefore, Δ*cbrA* behaved as a synthetic altruist (West et al. 2006). In contrast, the same assay conducted with a green-labeled wild-type as the actor showed that the wild-type helped the Δ*rhlA* recipient, but did so at no cost to itself (supplementary fig. S4, Supplementary Material online), which is consistent with the wild-type being metabolically prudent (Xavier et al. 2011; de Vargas Roditi et al. 2013).

In conclusion, we had genetically engineered a strain that overproduced rhamnolipids and grew slower in the nonpreferred nutrient conditions of swarming media. This strain, called Δ*cbrA*, did not swarm well but it could altruistically help rhamnolipid-deficient bacteria swarm.

### Experimental Evolution: Δ*cbrA* Is Rapidly Invaded by Spontaneous Mutants

We then carried out experimental evolution to determine the fate of the Δ*cbrA* altruist in swarming colonies. The experiments consisted of consecutive rounds of swarming where we allowed the bacteria to grow for 24 h, then washed the entire swarming plate and collect as much of the population as possible into a laboratory tube. Then, we quickly mixed the tube and used the mixture to inoculate a fresh plate for a next round of 24-h swarming (supplementary fig. S5, Supplementary Material online). *Pseudomonas aeruginosa* cells typically undergo approximately seven generations in each 24 h of swarming (Xavier et al. 2011).

**Fig. 2.**
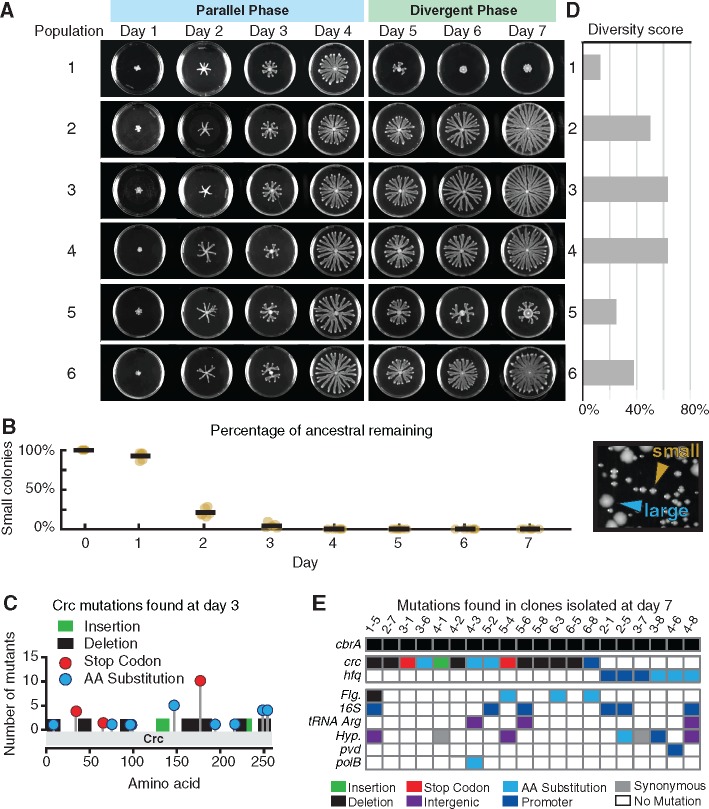
Experimental evolution starting with Δ*cbrA* showed rapid swarming recovery with suppressor mutations in either the *crc* or the *hfq* loci. (*A*) Left: The progression of swarming colony morphologies in the six replicate populations shown at the end of each 24 h of growth (1 through 3 and 4 through 6 were conducted in two separate dates). The first four days of the experiment were similar among the six populations and we termed this the “parallel phase.” The last three days were different and were termed this the “divergent phase” (see also supplementary fig. S6, Supplementary Material online). (*B*) The percentage of ancestral Δ*cbrA* colonies (ancestral remaining), estimated as the percentage of small colonies remaining (orange arrow head in inset image), decreased rapidly as suppressor mutants with larger colony size (blue arrow head in inset image) invaded each of the six populations. Whole genome sequencing of one large colony mutant revealed a mutation in *crc*. (*C*) Mutations revealed by targeted sequencing of the *crc* locus from 53 isolates from day 3 confirmed that loss of function in Crc suppressed the negative effects of Δ*cbrA*. (*D*) diversity scores calculated from isolates obtained from each population at day 7 (see supplementary fig. S7, Supplementary Material online). (*E*) Summary of mutations from 20 isolates from day 7 identified by whole genome sequencing. Every isolate had a mutation affecting either *crc* or *hfq* (full list in supplementary table S2, Supplementary Material online).

We performed this evolutionary experiment in two separate dates, each with three replicate populations leading to a total of six populations evolved in parallel (we first did populations 1–3 and then 4–6 at a later date). At the end of day 1, every population showed the weak swarming phenotype characteristic of Δ*cbrA*. However, between days 2 and 4, every population had recovered swarming remarkably well (fig. 2*A*). The dynamics were identical in the first four days and we termed this the “parallel phase.” The six populations diverged noticeably (fig. 2*A*) and measurably (supplementary fig. S6, Supplementary Material online) after day 4, with populations 1 and 5 reducing swarming while the others kept swarming. We termed that period after day 4 the “divergent phase.”

The rapid swarming recovery in the parallel phase was due to the fast invasion by suppressor mutants that compensated for the growth impairment of Δ*cbrA*. Those compensatory mutants stood out on hard agar plates (1.5% agar) because the Δ*cbrA* makes small colonies when plated in hard agar and these spontaneous mutants make larger colonies (fig. 2*B*). We sequenced the whole genome of one of those mutants and found a single point mutation: a two-nucleotide deletion in the gene *crc* (nucleotides 870–871). The spontaneous mutation in the *crc* locus possibly caused loss-of-function of Crc, and this could compensate for the Δ*cbrA* deletion because Crc is a master catabolite repressor downstream of CbrA/CbrB. The loss of CbrA/CbrB in Δ*cbrA* could be functionally de-repressing Crc (Sonnleitner et al. 2009). At day 3, the populations were practically all composed by large colony mutants; we isolated 53 of these isolates from all six populations and target-sequenced their *crc* locus. We found a range of *crc* mutations in >80% of them (fig. 2*C*) revealing that *crc* loss-of-function was a common solution to compensate the detrimental effects of Δ*cbrA*.

### Mutations in *Hfq*: A Rare but Better Alternative to *Crc* That Recovers Swarming

At the end of the experimental evolution—at day 7—the colonies varied widely (fig. 2*A*). We quantified the day 7 clonal diversity by first selecting 8 random isolates from each for a total of 48 isolates. Then, we measured a range of absorbance and fluorescence phenotypes (supplementary fig. S7A, Supplementary Material online) to group the isolates (supplementary fig. S7B, Supplementary Material online) and reduce the number down to 20 clonal representatives (supplementary fig. S7C, Supplementary Material online). This enabled us to calculate a diversity score for each population (fig. 2*D*); interestingly, the colonies with reduced swarming (1 and 5) had the lowest diversity.

We sequenced the whole genomes of these 20 isolates. As expected, all mutants harbored the *cbrA* deletion. We also found, interestingly, that each isolate had either a mutation in *crc* or a mutation related to *hfq* (fig. 2*E*). *hfq* encodes a molecular chaperone, Hfq, involved in the catabolite-repressive action of Crc (Moreno et al. 2012) and *hfq* mutations could be an alternative to *crc* to compensate for the Δ*cbrA* deletion. In contrast to *crc* mutants, *hfq* mutants did not have clear loss-of-function mutations (supplementary table S2, Supplementary Material online): The mutations were either amino acid substitutions in the *hfq* locus or alterations in the upstream DNA likely to be in its promoter. Quantitative PCR revealed that the isolates which had mutations upstream of the *hfq* locus (2-1, 2-5 and 3-7) decreased expression of *hfq* compared with the Δ*cbrA* control, but the isolates with amino acid substitutions inside the locus (3-8, 4-6, 4-8) maintained levels of expression of *hfq* (supplementary fig. S8, Supplementary Material online), suggesting that the mutations are either reducing Hfq activity through a decrease in its expression or a slight sequence modification.

We then sought to compare the effects of the two mutually exclusive types of suppressor mutations—in *crc* or *hfq*. We selected isolate 3-1 to represent ΔcbrA crc* mutants, as this isolate had no other detectable mutations. This ΔcbrA crc* recovered the growth rate of the wild-type (supplementary fig. S2A, Supplementary Material online), expressed less *rhlA* (supplementary fig. S2B, Supplementary Material online) and recovered swarming partially (supplementary fig. S9A, Supplementary Material online). We selected isolate 2-1 to represent ΔcbrA hfq* mutants because we only found one other mutation in its genome and its phenotype was qualitatively consistent with other ΔcbrA hfq* mutants isolated at day 7: They recovered the growth rate of the wild-type (supplementary fig. S2A, Supplementary Material online) and expressed more rhamnolipids than any ΔcbrA crc*, though less than the wild-type (supplementary fig. S2B, Supplementary Material online). Any of these *hfq*-mutated isolates also swarmed much better than the *crc*-mutated 3-1 (supplementary figs. S7C and S9B, Supplementary Material online).

### *Crc* and *Hfq* Mutants Rearrange the Perturbed Metabolome in Different Ways

**Fig. 3.**
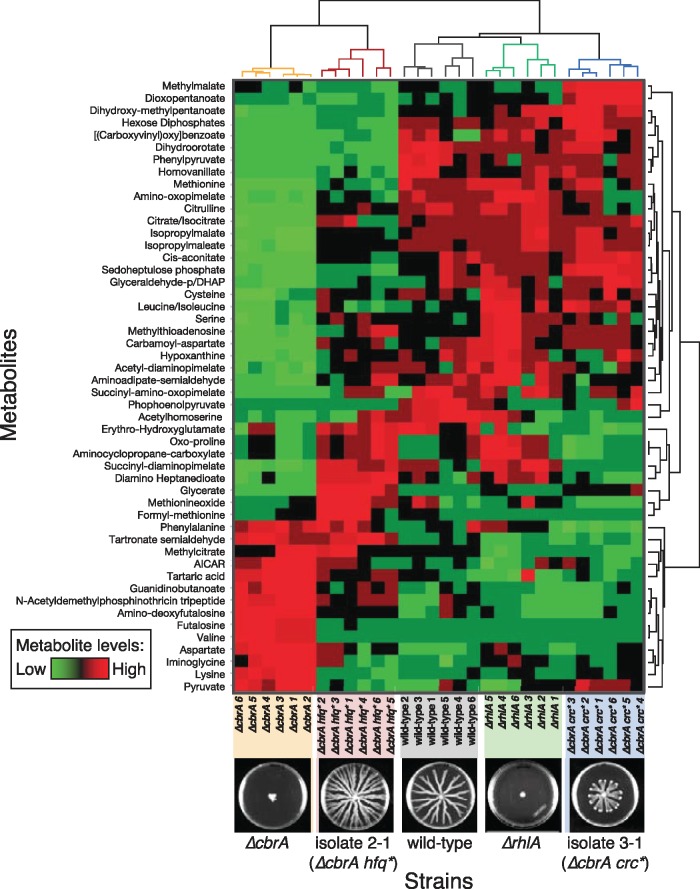
Metabolomics revealed distinct metabolic states in strains harboring *crc* or *hfq* mutations. The heat map shows levels of metabolites extracted from exponential phase *Pseudomonas aeruginosa* mutants after growth in synthetic glycerol media (methods). Metabolites were identified by LC-MS (each row was normalized to mean 0 and standard deviation of 1; red means above average, green means below average and black means close to average levels). Each row corresponds to a metabolite and each column represents a technical replicate from each strain (six replicates total for each strain, the strains shown in order are Δ*cbrA*, isolate 2-1 (a ΔcbrA hfq*), wild-type, Δ*rhlA* and isolate 3-1 (a ΔcbrA crc*). The hierarchical clustering of strains is shown on top and the hierarchical clustering of metabolites is shown on the right. Importantly, isolate 2-1 clustered together with Δ*cbrA*, not the wild-type, whereas isolate 3-1 clustered with the wild-type and the Δ*rhlA* strain.

**Fig. 4.**
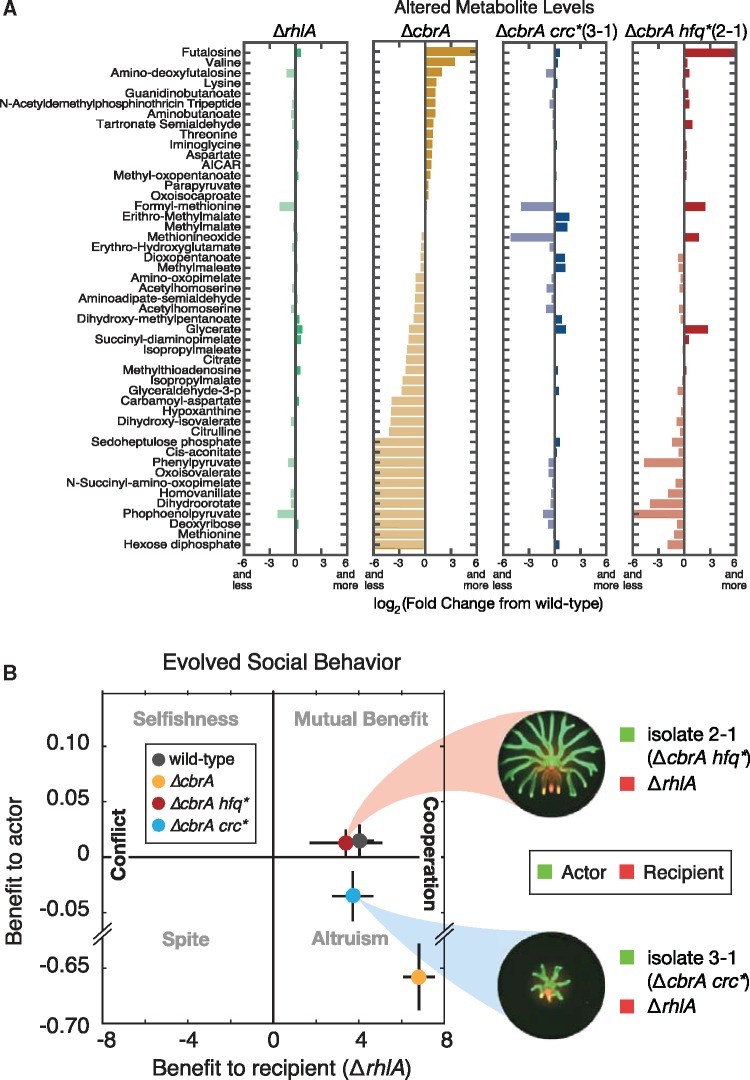
The metabolome of ΔcbrA hfq* mutant 2-1 showed incomplete recovery but its social behavior resembled the wild-type. (*A*) Differences in levels of metabolites significantly altered between wild-type and Δ*rhlA*, Δ*cbrA*, isolate 3-1 (ΔcbrA crc*) or isolate (ΔcbrA hfq*). (*B*) Left: Quantification of social behavior assays for isolate 3-1 and 2-1*.* The 3-1, the ΔcbrA hfq* mutant, helped Δ*rhlA* without a cost and should be favored by natural selection whereas 2-1, the ΔcbrA crc*, retained a partial cost (net negative benefit) and would be disfavored by natural selection. Right: actor-recipient assays performed for isolates 3-1 and 2-1 mixed with the Δ*rhlA* nonswarmer.

We then investigated the underlying changes in metabolism linked with the different swarming phenotypes. We grew the strains at 37°C in a fully defined liquid media with glycerol as the sole carbon source and ammonium nitrate as the nitrogen source. We extracted the intracellular metabolites we used high-throughput LC-MS (see methods) to quantify and compare the metabolomes of the wild-type, Δ*rhlA*, Δ*cbrA*, and the mutants 3-1 (ΔcbrA crc*) and 2-1 (ΔcbrA hfq*) (fig. 3*A*, all strains contained P_*rhlAB*_-*gfp* genomic reporters). We built a clustergram using hierarchical clustering of the metabolomics data obtained from six replicates for each strain (fig. 3) and we analyzed the subset of metabolites most significantly changed among strains (fig. 4*A*). This analysis led to four main conclusions: First, deleting *rhlA* did not perturb the metabolome significantly as seen by the close clustering of the Δ*rhlA* and the wild-type (fig. 3) and its levels of metabolites, which remain very similar to the wild-type levels (fig. 4*A*, first panel). This supports previous revelations that *P. aeruginosa* regulates intracellular carbon to ensure homeostasis whether it produces rhamnolipids or not (Boyle et al. 2015). Second, *cbrA* is necessary for that homeostasis and perturbing it altered the metabolome (fig. 4*A*, second panel). This finding was expected from the broad influence of CbrA/CbrB on metabolic regulation (Li and Lu 2007; Sonnleitner et al. 2009; Yeung et al. 2011; Zhang et al. 2015), but the effect on the metabolome had not been directly investigated before. Third, mutant 3-1 harboring the suppressive mutation in *crc* recovered the wild-type metabolome as the vast majority of metabolites perturbed in Δ*cbrA* returned to wild-type levels (fig. 4*A*, third panel). Finally, mutant 2-1 harboring the suppressive mutation in *hfq* recovered some of the wild-type metabolite levels, but most of the metabolome remained surprisingly similar to the Δ*cbrA* strain (fig. 4*A*, fourth panel). The alternative metabolome recovery—seen also in the clustering of the Δ*cbrA* with ΔcbrA hfq* in the upper dendrogram (fig. 3)—was unexpected since this mutant has a swarming phenotype more similar to the wild-type (supplementary fig. S9B, Supplementary Material online).

The two mutants investigated, 3-1 (a ΔcbrA crc*) and 2-1 (a ΔcbrA hfq*) behaved differently according to the actor-recipient assay: 3-1 had a reduced cost of swarming relative to the Δ*cbrA* strain, but did not recover completely; 2-1 reduced cost entirely (fig. 4*B*). These results suggested that the two mutually exclusive mutation types—in *crc* or *hfq*—could determine divergent paths to recover from the Δ*cbrA* loss of metabolic prudence. The mutations in *crc* and *hfq* were the only pattern common among all mutants, but some of the non-*crc*/*hfq* mutations found could be themselves linked to metabolism (e.g., *dcd*, *fab*, pyr tRNA synthesis). These mutations suggest there is room to adapt the metabolism further in swarming evolution. Nonetheless, the results suggest that while the most common mutation type, *crc*, invaded rapidly to reach >80% at day 3, this may not have been the best solution. Mutations in *hfq*, though less common, seemed to rearrange the metabolome differently, regaining a higher *rhlA* expression regardless of additional mutations (supplementary fig. S2B, Supplementary Material online) and, at least in 2-1, could recover the low-cost social behavior of the metabolically prudent wild-type (fig. 4*B*).

### Immotile Mutants Blocked Swarming in Populations 1 and 5

**Fig. 5.**
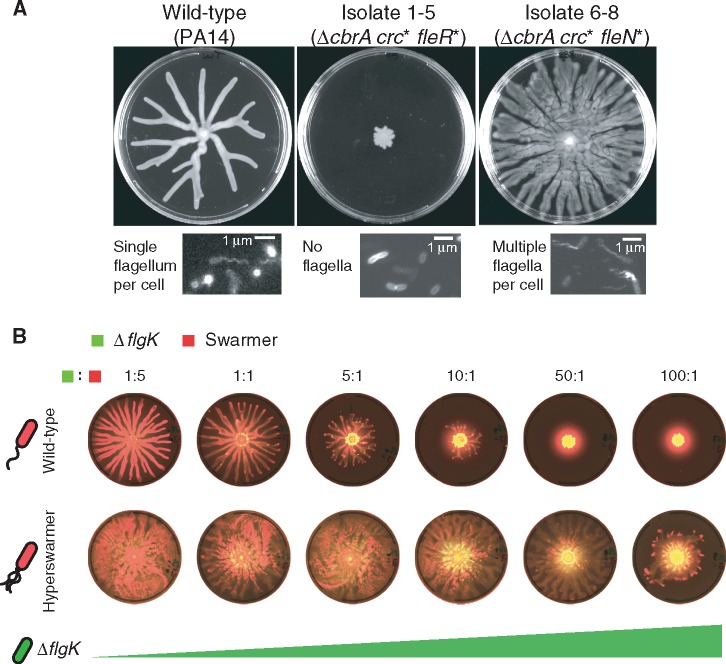
Flagella-less mutants isolated from two *crc-*mutated lineages block wild-type swarming but multi-flagellated hyperswarmers are less vulnerable to blocking. (*A*) Swarms of wild-type, ΔcbrA crc* fleR* (isolate 1-5), and ΔcbrA crc* fleN* (isolate 6-8) with insets detailing labeled flagella. Isolate 1-5 does not have flagella while 6-8 has multiple flagella (the wild-type is mono-flagellated; scale 5 µm; see also supplementary fig. S10, Supplementary Material online). (*B*) Swarming competitions between fluorescently labeled wild-type, wild-type background hyperswarmer and wild-type background Δ*flgK*. Mixed colonies at 5:1 Δ*flgK*:wild-type mixing ratio show decreased swarming whereas the hyperswarmer, less vulnerable to blocking, only shows decreased swarming radius at 100:1 Δ*flgK*:hyperswarmer.

The mutually exclusive *crc* and *hfq* mutations were a major factor in the evolution of swarming recovery but how did they explain the divergent phase? We detected *hfq* mutants only in three of the six populations—2, 3 and 4—and all of those swarmed robustly up to day 7. Of the other three, two populations—1 and 5—severely reduced their swarming size whereas population 6 swarmed robustly (fig. 2*A* andsupplementary fig. S6, Supplementary Material online). The genomes sequenced at day 7 suggested that this variation in swarming could be explained by the mutations in flagella genes (fig. 2*E* and supplementary table S2, Supplementary Material online). Isolates 1-5 and 5-4, which both harbored *crc* mutations, had additional mutations in *fleR* and *fleQ*; these isolates lacked flagella (fig. 5*A*, center) and were incapable of swarming (supplementary fig. S10, Supplementary Material online). Intriguingly, these nonswarming isolates blocked the wild-type in swarming competitions (supplementary fig. S10, Supplementary Material online) and their emergence could explain the reduced day 7 swarming in populations 1 and 5.

The remaining population—population 6—had two *crc* mutants, 6-3 and 6-8, with additional mutations in flagella-related loci, yet the population swarmed robustly. The difference between this and population 1 and 5 was that the flagella mutations of 6-3 and 6-8 were in *fleN* (fig. 2*E* and supplementary table S2, Supplementary Material online). Mutations in *fleN* are known from previous work (van Ditmarsch et al. 2013; Deforet et al. 2014) to turn bacteria into multiflagellated hyperswarmers (fig. 5*A*, right panel and supplementary fig. S10, Supplementary Material online). Swarming competitions revealed that in fact 6-3 and 6-8 were both immune to blocking by the flagella-less 1-5 or 5-4 in conditions that blocked wild-type swarming (supplementary fig. S10, Supplementary Material online).

To better determine whether loss of flagella was sufficient to block swarming and whether *fleN*-mutated hyperswarmers were immune to blocking, we carried out competitions using the wild-type, an engineered flagella-less strain (Δ*flgK*) and a *fleN*-mutated hyperswarmer (FleN(V178G)) that we had evolved previously (van Ditmarsch et al. 2013). The Δ*flgK* strain could indeed block the wild-type in swarming competitions starting as low as 5:1 (fig. 5*B*, top row and supplementary video S2, Supplementary Material online). The hyperswarmer was less prone to blocking and required a 100:1 starting advantage of Δ*flgK* for any noticeable effect (fig. 5*B*, bottom row). These experiments confirmed that flagella-less mutants could indeed block others from swarming and that hyperswarmers were resilient to blocking, explaining the why populations 1 and 5—but not population 6—suffered swarming reductions at day 7.

## Discussion

We showed that genetic perturbation of a key metabolic regulator, *cbrA*, profoundly altered the metabolome of *P. aeruginosa* affecting the evolution of its social behavior. The genetic excision executed in this catabolite repressor produced bacteria that grew slower on casamino acid media, expressed higher levels of public good rhamnolipids and could not swarm by themselves but, altruistically, helped others swarm.

Experimental evolution revealed a rapid recovery of social behavior. First, we observed a rapid expansion of *crc* mutants in all populations. *crc* were >80% of the mutants at day 3, indicating their important role in the dynamics of the parallel phase. Whole genome sequencing of 20 isolates from the final day of the experiment revealed an alternative path to *crc* mutations because each mutant had a mutation in one of two loci downstream of *cbrA*—*crc* or *hfq*. *crc* mutations were still the majority (in 14/20) and one possible explanation is that the *crc* locus is longer: 787 bp compared with the 251 bp of *hfq* (found in 6/20). Perhaps more importantly, the mutations found in *crc* show that a loss-of-function at that locus was sufficient to recover from the slow growth of Δ*cbrA*, and there were many possibilities for mutations to cause loss-of-function. In *hfq*, we only found amino acid substitutions and mutations in the promoter region suggesting a reduction of Hfq expression or reduction of Hfq activity, but not Hfq loss-of-function. Mutations in *hfq* were less frequent, possibly because the chaperon Hfq has many other functions (Sonnleitner et al. 2006); there should be fewer possibilities for *hfq* to mutate and suppress the effect of Δ*cbrA* without deleterious pleiotropic effects.

We had previously experimented with swarming evolution starting from wild-type bacteria; those experiments led to a remarkably parallel evolution of mutliflagellated hyperswarmers (van Ditmarsch et al. 2013) and provided new insights on the regulation of bacterial flagella (Kearns 2010). Those experiments had started with a metabolically prudent wild-type and thus taught us little on the role of metabolism in swarming behavior. Here, starting with an engineered strain lacking proper metabolic prudence—the Δ*cbrA* strain—provided a unique view on how the internal metabolic state of an organism changes while it recovers from a perturbed social behavior. We saw that *crc* mutants recovered the wild-type’s metabolomic state and its growth rate, but retained a fitness cost of swarming behavior. *hfq* mutants recovered social behavior similar to the wild-type even if their metabolome remained perturbed. This unexpected trend suggests that in order to recover low-cost social behavior in the absence of proper metabolic regulation organisms may need to reorganize their metabolome.

Hfq is an abundant regulator of with many physiological roles, which makes it harder to determine exactly how *hfq* mutations compensate for the fitness cost of swarming cooperation. Despite its metabolome being more similar to Δ*cbrA* than to the wild-type, some metabolites—including valine, lysine, citrulline and citrate—did recover wild-type levels in *hfq*. The connection between these metabolites and swarming fitness is unclear, but they provide targets for future investigation into the mechanism of metabolic prudence. A deeper study of the pleiotropic effects of *hfq* on the metabolome could also shed light on why *hfq* mutations affect some metabolites and not others, and on the role of Hfq in the ability of bacteria to divert resources away from central metabolism and into public goods essential for social behavior.

We also saw that while all six populations recovered swarming by day 4, by day 6, two populations decreased swarming again. Those two populations contained *crc*-mutated lineages with additional mutations in flagella genes that caused loss of flagella motility, and those mutants blocked others from swarming. The emergence of flagellar mutations after recovery of growth rate by mutations in either *crc* or *hfq* suggests that after an initial competition for metabolic advantage, the evolution centers again on competition between motility strategies, which we had observed previously in the evolution of hyperswarmers (van Ditmarsch et al. 2013). Interestingly, here we found two *crc*-mutated isolates from population 6 had mutations in *fleN* that turned them into hyperswarmers and were immune to blocking. That population continued swarming robustly until the end of the experiment.

Bacteria have many social behaviors besides swarming—they communicate by quorum sensing, produce extracellular polymers to form biofilms, and share nutrient scavenging molecules (Crespi 2001; West et al. 2006)—and they can implement sophisticated social strategies even without having a brain (Axelrod and Hamilton 1981). Bacteria rely on these social interactions but they live in chaotic worlds of ever-changing environments and random encounters with other strains and species (Nadell et al. 2013). Horizontal gene transfer (Niehus et al. 2015), frequent mutants (Rainey and Rainey 2003; Boles et al. 2004; Kim et al. 2014; Flynn et al. 2016), dispersal, and environmental mixing (Griffin et al. 2004; Nadell et al. 2013) constantly alter genetic relatedness and bacteria cannot always count on a stable social structure. Evolution should favor strategies that regulate social behaviors and lower their metabolic cost to the extent possible (Xavier et al. 2011; Dandekar et al. 2012; Gupta and Schuster 2013).

Beyond bacteria, nature shows an astounding diversity of social behaviors (Robinson et al. 2008). Wolfs hunt in packs, termites build colonies of thousands, even plants communicate with chemicals secreted from their roots (Bais et al. 2004). Can we ever identify general principles among them? Our experiments suggest a common premise: metabolism is the currency of all physiological processes and its influence on social behavior is likely conserved in all species, including humans. Proteins are the products of genes and they control metabolism directly, which make metabolic regulation a proximate mean for genes to command social behavior. All else being equal, natural selection should favor expression of a social behavior only when its metabolic cost is low, which closes the loop from genes to social behavior (fig. 1*A*). Genetic tools to manipulate the metabolism of animals already exist (Robinson et al. 2008; LeBoeuf et al. 2013) but it would be difficult to investigate the role of metabolism on the evolution of animal social behavior with the same detail as we did here in bacteria. Experimental evolution requires many generations and even the quick *C. elegans* can only reproduce once every ∼3.5 days. Bacteria have generations of <1 h, small genomes and large population sizes with billions of organisms which make them unique models for experimental testing of evolution theories (Lenski et al. 1991; Fiegna et al. 2006; van Ditmarsch and Xavier 2011; Lee and Marx 2012; Lind et al. 2015; Kim et al. 2016). Understanding how metabolism affects social behavior can ultimately help us explain how organisms—ourselves included—govern their behavior towards others. And it could open the door to explain why factors that alter metabolism like the gut microbiomes of animals (Turnbaugh et al. 2006; Ezenwa et al. 2012; McFall-Ngai et al. 2013) can influence social behavior.

## Materials and Methods

### Deletion in *cbrA* Locus

All primers used were obtained from IDT (Coralville, Iowa) unless otherwise stated. All chemical reagents came from Fisher Scientific (Waltham, Massachusetts) unless otherwise stated.

Primer sequences:

Upstream sequence for deletion:

UpCbrAFwd: GGGGACAAGTTTGTACAAAAAAGCAG GCTACAGCTGGAATATCGCTTCGTCG

UpCbrARev: CGCCTTCTCGCTGGTGATCCGTCAGCATCGCGCTGGCTC

Downstream sequence for deletion:

DownCbrAFwd: GATCACCAGCGAGAAGGCG

DownCbrARev: GGGGACCACTTTGTACAAGAAAGCTGGGTACAGCTCGGATTCGATCAGGG

PCR primers placed outside the area of genomic manipulation to amplify the region and confirm deletion:

CbrAPCRFwd: AAGCATGAGGGAAGCGGCAT

CbrAPCRRev: AACAACATGTCGAAGGAGGG

(The deletion in the *cbrA* locus was also confirmed by the multiple sequencing of Δ*cbrA* mutants evolved by experimental evolution. Every mutant harbored the correct deletion).

Sequencing primers to sequence the region after PCR:

CbrASeqFwd: CCTCATCGTGGTCACTTTC

CbrASeqRev: ATTCAGCTCTCTCGACGTGC

See procedures in Shanks et al. (2006) and van Ditmarsch et al. (2013) for additional details. Briefly, Milli-Q-Water-resuspended stocks of oligonucleotides (100 μM) were stored at −20 °C. Stock solutions were diluted 10-fold to a working concentration. Regions of homology outside of the *cbrA* locus (PA14_62530) were created using for upstream region UpCbrAFwd and UpCbrARev, and for downstream region DownCbrAFwd and DownCbrARev. The deletion construct was obtained through splicing overlap extension (SOE) PCR, followed by Gateway cloning (Life Technologies, Grand Island, NY) before integration of the suicide plasmid into the *P. aeruginosa* genome. SOE was done using UpCbrAFwd and DownCbrARev. The transformation yielded a scarred deletion encoding a 27AA peptide. Integration of the suicide plasmid into the genome of *P. aeruginosa* was verified with primers CbrAPCRFwd and CbrAPCRRev. The isolates of interest that were obtained after sucrose counter-selection were verified through PCR followed by sequencing using CbrAPCRFwd, CbrAPCRRev, CbrASeqFwd and CbrASeqRev. The intended deletion was also confirmed independently by each of the 20 whole genomes sequences: each of these strains derived from Δ*cbrA* contained a 2,868 bp deletion in the *cbrA* locus (supplementary table S1, Supplementary Material online).

### Media

Casamino acids minimal media nutrient composition was used in liquid media and swarming plates prepared with 800 ml of Milipore water, 200 ml of 5× minimal salts buffer, 1 ml of 1 M magnesium sulphate, 0.1 ml of calcium sulphate, 25 ml of 200 g/l solution of casamino acids (Bacto TM from BD, Sparks, MD). 5× stock minimal salts buffer were prepared with 12 g of Na_2_HPO_4_ (anhydrous), 15 g of KH_2_PO_4_ (anhydrous) and 2.5 g of NaCl into 1 l water. Synthetic media for liquid culture used in metabolomics assays was prepared with 800 ml of Milipore water, 200 ml of 5× minimal salts buffer, 1 ml of 1 M magnesium sulphate, 0.1 ml of calcium sulphate with stated concentrations of carbon (glycerol), nitrogen (ammonium sulphate) and iron at 5 μM (iron III sulphate). 1.5% agar was used for hard agar plates, and 0.5% agar for swarming plates.

### Swarming Assay

Swarming assays were performed as described previously (Xavier et al. 2011; van Ditmarsch et al. 2013). Briefly, we used casamino acids media 0.5% agar plates; strains were grown in 3 ml LB overnight cultures at 37°C. About 1 ml of each strain was washed 2× with phosphate-buffered saline (PBS) and 2 μl of the final suspension was used to inoculate a swarming plate by spotting this volume on the surface of the agar plate without penetrating the agar with the pipette tip; finally, plates were incubated at 37°C for 24 h. For co-inoculated swarms, where swarming plates were inoculated with two strains that were not well mixed (e.g., fig. 1*C*), 2 μl of the cell suspension for each strain were spotted onto the surface of the agar plate 2 mm apart.

### Imaging

Swarming colonies were imaged using a Typhoon 9400 (GE Healthcare) for fluorescent images and a Chemidoc gel doc imager (Bio Rad) for black-and-white images.

### Growth Rate

Growth curves were performed as reported previously (van Ditmarsch and Xavier 2011) in casamino acids media. Strains were inoculated into 3 ml of LB and incubated overnight at 37°C with shaking. About 1 ml of overnight culture was pelleted at room temperature and washed twice with 1 ml PBS and then inoculated into minimal media at an OD of 0.0025 in a BD Falcon (BD Biosciences, San Jose, CA) 96 well flat-bottom plate with 150 µl of media per well. The plate was incubated at 37°C in a Tecan Infinite M1000 or Tecan Infinite M1000 Pro plate reader (Männedorf, Switzerland) with and orbital shaking of 4 mm amplitude. Optical density was measured at 600 nm. Measurements were taken every 10 min. Exponential growth rates were calculated from the first 10 time points occurring after the culture had reached an OD of 0.01 on Tecan (Männedorf, Switzerland) plate reader.

### *rhlAB* Expression

*rhlAB* expression was measured using a P*_rhlAB_-gfp* promoter originally created from cloning the upstream region of the *rhlA* locus in the *P. aeruginosa* PAO1 strain. The cloned fragment is 628 bp and extends from 551-bp upstream of the *rhlA* translational start and is contained in a plasmid called pYL122 (Lequette and Greenberg 2005). We previously used this plasmid to introduce the construct into PA14 (Xavier et al. 2011) using a variation of the insertion protocol introduced by Hoang et al. (1998) which places the construct into the *P. aeruginosa* genome at the locus *pabC* (PA14_25710). The 628-bp sequence, which was originally taken from PAO1 (Lequette and Greenberg 2005), differs from the PA14 native sequence in four nucleotides (supplementary table S3, Supplementary Material online). qPCR analysis validated that despite these four nucleotide differences the expression of *gfp* matched well with native expression of *rhlA* (supplementary fig. S1, Supplementary Material online). GFP measurements were taken at the time indicated from cultures inoculated into minimal media at an OD of 0.0025 in a BD Falcon (BD Biosciences, San Jose, CA) 96-well flat-bottom plate with 150 µl of media per well. The plate was incubated at 37°C in a Tecan Infinite M1000 or Tecan Infinite M1000 Pro plate reader (Männedorf, Switzerland) with and orbital shaking of 4 mm amplitude.

### RNA Isolation and qPCR

An initial culture was inoculated in LB liquid media and incubated overnight at 37°C. The cultures were then passaged to fresh casamino acids media and incubated at 37°C into exponential phase. For analysis of *gfp* and *rhlAB* levels, cells were harvested from beginning to late exponential phase to confirm the fidelity of *gfp* to *rhlAB* at different points in exponential phase for the wild-type strain. For analysis of *hfq*, all strains were harvested at the same time. Cells were pelleted from 1 ml of exponential phase liquid culture in casamino acids media at 4°C and the supernatant was removed on ice. The pellet was gently dissolved in 1 ml TRIzol (Fisher Scientific). The solution was transferred to a Phase Lock tube, 400 μl of chloroform was added and the tubes were shaken vigorously for 10 s. The solution was then incubated at room temperature for 5 min and then centrifuged for 15 min at maximum speed on a bench top centrifuge. The aqueous phase was transferred to a fresh 1.5 ml tube (Eppendorf), 450 μl of isopropanol were added to each tube and the tube was mixed immediately. The solution was incubated for 30 min at room temperature before being centrifuged at maximum speed in a bench top centrifuge. The supernatant was discarded and the pellet was washed with 350 μl of 70% ethanol. Then, the tubes were centrifuged for 10 min at room temperature at maximum speed. The supernatant was discarded and the pellet was dried and re-suspended in 50 μl RNase-free water. Quantitative PCR was performed using the isolated RNA and the OneStep RT-PCR kit (Qiagen). The error bars for the fold change of *hfq* from the wild-type represent 95% confidence intervals calculated using the *fitlm.m* function in Matlab.

qPCR Primers:

*proC* (housekeeping gene used as reference):

proC Fwd: GTG GTC CTG TCG GTC AAGproC Rev: GAT GGA GAC GAT CAG TTG CTC

*hfq*:

hfq Fwd: GAC CCT TAC CTC AAT ACC CTGhfq Rev: GCG TGC TTG TAA ACC ATC TG

*gfp*:

gfp Fwd: GAT GGT GAT GTT AAT GGG CACgfp Rev: GGG TAA GTT TTC CGT ATG TTG C

*rhlA*:

rhlA Fwd: GGC GCG AAA GTC TGT TGG T

rhlA Rev: CCA ACG CGC TCG ACA TG

### Experimental Evolution

Experimental evolution was performed as described previously (van Ditmarsch et al. 2013). To initiate experimental evolution the Δ*cbrA*:P*_rhlAB_-gfp* strain was inoculated in 3 ml of LB and incubated overnight at 37°C. About 1 ml of overnight culture was washed 2× with PBS and 2 μl of the final suspension was used to initiate each of three replicates (we repeated the experiment, for a total of six replicates) on swarming minimal media agar plates. Each of the swarms grown after 24 h was then washed from the plate into a volume of 3 ml 1× PBS. About 2 µl of this cell suspension were then used to inoculate a fresh swarming plate, which was then incubated for 24 h at 37°C. The washing and inoculation of a fresh plate was repeated for each day of the experimental evolution (supplementary fig. S5, Supplementary Material online).

### Targeted Sequencing of *crc*

PCR Primers:

CRC PCR Fwd: CTT CCT TGC GGT TGAAGC AC

CRC PCR Rev: CTG GTG GTG ATC GGC TTC TT

Sequencing Primers:

CRC SEQ Fwd 1: AGA AAT AGG GGC TGG TGC

CRC SEQ Fwd 2: TTC GGC AAC CTC GGC TAT G

CRC SEQ Rev: CAA ACG GCT GTA GAG TGC

CRC PCR Fwd and CRC PCR Rev were used to amplify the *crc* gene. Sequencing was then performed using the CRC sequencing primers.

### Liquid Culture Measurements

Strains were inoculated into 3 ml of LB and incubated overnight at 37°C. About 1 ml of overnight culture was pelleted at room temperature and washed twice with 1 ml PBS and then inoculated into minimal media at an OD of 0.0025 in a BD Falcon (BD Biosciences, San Jose, CA) 96-well flat-bottom plate with 150 µl of media per well. The plate was incubated at 37°C in on a Talboys Advanced Digital 1000-3 Orbital Shaker (Thorofare, NJ). The plates were shaken and incubated for 24 h. Absorbances and florescence emissions were measured using the Tecan (Männedorf, Switzerland) plate reader. Absorbance measurements were done at 600, 690, and 310 nm. GFP was measured using an excitation of 488 nm of and an emission of 525 nm. Pyoverdine (pvd) was measured using an excitation of 405 nm and an emission of 465 nm. A measurement with an excitation of 340 nm and an emission of 440 nm was also performed to help discriminate between the isolates.

### Social Behavior Assay

Strains were grown and washed according to the swarming assay described earlier. Before inoculation Δ*rhlA* P_A1/04/03_-DsRed (constitutively expressing a red fluorescent protein) was mixed with the actor strain at a ratio of 1:25 Δ*rhlA* strain to actor strain (see detailed list of strains used in supplementary table S1, Supplementary Material online). 2 μl of the mixed cell suspension were used to inoculate the swarming plate. After 24 h of incubation the swarms were washed and the cells collected in 3 ml 1× PBS. The collected cellular suspension was used for colony forming unit plating on hard agar plates. Colony forming units (CFU) were used to determine the ratio and total number of cells for each strain. The benefit to the actor is calculated by the following equation: *(Starting actor fraction)−(Final actor fraction)*. The fraction of the actor strain is determined by: *(number of actor cells/total cells observed)*. The number of cells is determined by CFU count. The benefit to the DsRedExpress labeled Δ*rhlA* recipient is calculated by: *(ΔrhlA total cell number when mixed with actor/ΔrhlA total cell number when mixed with GFP labeled ΔrhlA)*. Data shown was always from three biological replicates with three technical replicates each for every actor-recipient pair for a total sample size of 9.

### Diversity Score

Liquid culture measurements for each strain were used to generate a dendrogram in Matlab. A linkage distance cutoff of 1.12 was used to set the number of clusters present. The diversity score was then calculated for each population by the following formula: (number of clusters represented in population/8)×100 yielding a maximum possible score of 100% and a minimum possible score of 12.5%.

### Whole Genome Sequencing

Strains were inoculated into 3 ml of LB and incubated overnight at 37°C. Genomic DNA extraction was performed using Wizard Genomic DNA Purification Kit (Promega). Genomes were sequenced at the New York Genome Center with 2× 125-bp HiSeq with coverage of 50×. Mutations were determined using Breseq (Deatherage and Barrick 2014), and we used the genome NCBI Reference Sequence: NC_008463.1 corresponding to strain *Pseudomonas aeruginosa* PA14 to align the sequences. Whole genome sequencing analysis by Breseq is subject to random calling of mutations in the P_*rhlAB*_-*gfp* region (supplementary table S2, Supplementary Material online), and the calls which are trivial artifacts of the reporter fusion were excluded from further analysis as well as from the summary table in figure 2*E*.

### Metabolite Extraction

Strains were grown to exponential phase in fully synthetic minimal media with 3.0 gC/l (glycerol) 0.5 gN/l (ammonium sulfate) and 5 µM iron (iron III sulfate). Bacteria were loaded onto 0.20 µm nylon membranes (Millipore) using a vacuum apparatus. Bacteria laden filters were immediately transferred to pre-warmed hard agar plates with a media composition identical to the synthetic liquid media. Filters on hard agar plates were incubated at 37°C for 2.5 h. Filters were then transferred to 35 mm polystyrene dishes (Falcon) with 1 ml 2:2:1 acetonitrile:methanol:water quenching buffer on dry ice. Filters were incubated in quenching buffer for 15 min on ice and cells were removed from filters by scraping. Quenching buffer containing cell lysate was then transferred to 1.5 ml micro-centrifuge tubes (Eppendorf) on ice and centrifuged at 16,000 rcf for 10 min at 4°C. Supernatant was then transferred to a fresh tube and stored at −80°C. The experiment was performed with two biological replicates with three technical replicates each.

### Metabolomics Analysis

LC-MS based metabolomic profiling was utilized to report the metabolic landscapes of the wild-type and *Pseudomonas aeruginosa* isogenic mutants, all carrying the P_*rhlAB*_-*gfp* reporter fusion. As shown previously (Hosni et al. 2011), extracted metabolites were separated on a Cogent Diamond Hydride type C column (MicroSolv). The mobile phase comprises solvent A (ddH_2_O with 0.2% formic acid) and solvent B (acetonitrile with 0.2% formic acid). The gradient used was as listed below: 0–2 min, 85% B; 3–5 min, 80% B; 6–7 min, 75%; 8–9 min, 70% B; 10–11.1 min, 50% B; 11.1–14 min 20% B; 14.1–24 min 5% B followed by a 10-min reequilibration period at 85% B at a flow rate of 0.4 ml/min. Agilent Accurate Mass 6220 TOF coupled to an Agilent 1200LC system was used as the mass spectrometer. The metabolites were identified by accurate mass matching (mass tolerance < 0.005 Da) and where available retention time matching against chemical standards. Metabolite ion counts were extracted using Profinder 8.0 and Qualitative Analysis 6.0 (Agilent). Targeted analysis was performed to identify metabolite levels in each strain. Putative metabolites were identified based on accurate mass (m/z). We include the peak areas in the supplementary file metabolomics.xlsx, Supplementary Material online.

In order to correct for variation in sample concentration, we calculated the scaling factor by fitting a generalized linear model using the *fitglm.m* function in Matlab using log as the link function with the following formula in Wilkinson notation:

Log(peakArea) ∼ mutant_id * experiment_id + metabolite_ id.

This calculated the scaling coefficient under the assumption that majority of metabolites are unchanged. We selected metabolites that did change between strains by defying those that passed multiple hypothesis correction using ANOVA with an alpha of 0.01 and an *n* of 142.

### Flagella Staining

Flagella staining was adapted from (Darnton et al. 2007) performed as reported previously (Deforet et al. 2014). An initial culture was inoculated in LB liquid media and incubated overnight at 37°C. The cultures were then passaged to fresh LB media and incubated at 37°C into exponential phase. Cells in exponential phase in LB liquid media were gently washed 3 times in 1 ml 1× PBS (centrifuged at 350 rcf for 10 min). A solution of 10 mg/ml (suspended in dimethyl sulfoxide) Alexa Fluor 488 carboxylic acid, succinimidyl ester (Molecular Probes) was added to the cellular suspension at a ratio of 1:50 (label solution volume:cell suspension volume). Cells were incubated with the label for 90 min at room temperature, washed 3 times in 1× PBS (centrifuged at 350 rcf for 10 min) and suspended in 1× PBS to a final OD600 of ∼0.01.

## Supplementary Material

Supplementary data are available at *Molecular Biology and Evolution* online.

## Supplementary Material

Supplementary Data
