# Supplementary material for: Metabolism and the Evolution of Social Behavior
Source: Mol Biol Evol. 2017 Jun 8;34(9):2367–79. doi: 10.1093/molbev/msx174 (PMC5850603; doi:10.1093/molbev/msx174)
Supplement: Supplementary Data [file msx174_Supp.zip › Supplemental Material_5_20_2017.docx]

**Supplemental Figures and Tables**


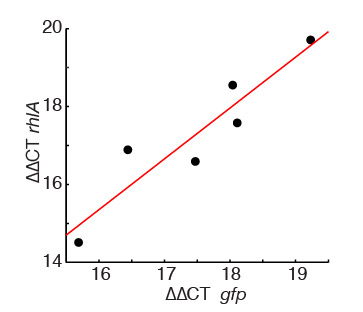


**Figure S1. Expression of *gfp* transcript driven by the reporter fusion P*_rhlAB_-gfp* correlates with *rhlA* expression which is driven by the native P*_rhlAB_*.** Quantitative PCR results comparing *rhlA* and *gfp* levels for bacteria harvested at different times during exponential phase, during which bacteria increase their expression of the rhamnolipid synthesis operon *rhlAB*. *rhlA* transcript levels correlate with the level of *gfp* transcript levels, justifying our use of GFP expression as a reporter for *rhlA*.


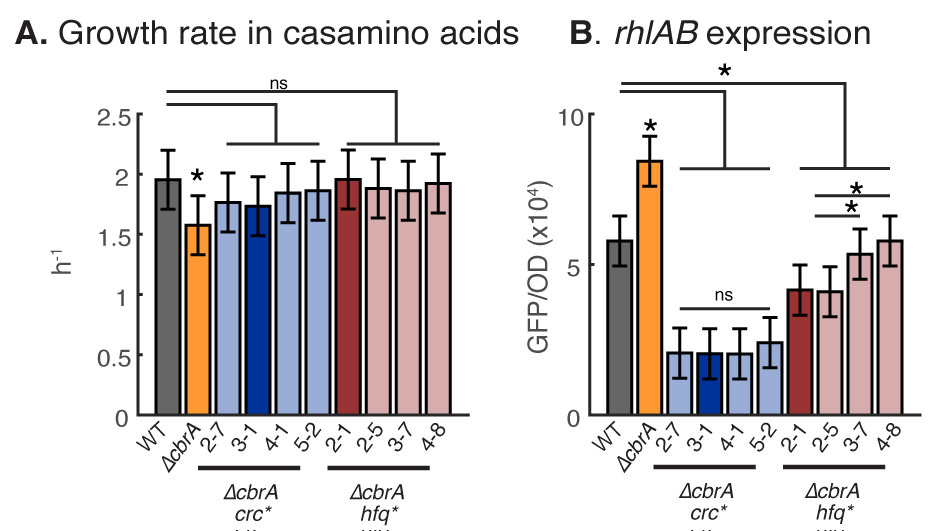


**Figure S2. Growth rates and rhamnolipid synthesis expression in ∆*cbrA* and the kinds of suppressive mutants in *crc* and *hfq* (four of each kind)*.* A**. Exponential growth rate of strains measured liquid casamino acids media prepared with the same nutrient concentration as swarming plates. The ∆*cbrA* engineered strain grew significantly slower than the wild-type (p<0.05) while isolates harboring ∆*cbrA crc** mutations (2-7, 3-1, 4-1 and 5-1) and isolates harboring ∆*cbrA hfq** mutations (2-1, 2-5, 3-7 and 4-8) showed no difference from wild-type p>0.05). **B.** *rhlAB* expression measured from the P*_rhlAB_*-*gfp* promoter (see methods). ∆*cbrA* over expressed *rhlAB* while ∆*cbrA crc** and ∆*cbrA hfq** mutants under-expressed *rhlAB.* There was no detectable difference in growth rate or *rhlAB* expression between isolate 3-1—the isolate chose as a representative ∆*cbrA crc** mutant—and the ∆*cbrA crc** mutants, 2-7, 4-1 and 5-2. Likewise, There was no detectable difference in growth rate expression between isolate 2-1 and the other ∆*cbrA hfq** mutants. There was a small, but detectable, quantitative difference in rhlAB expression between 2-1 and 2-7 and 3-8, but the quantitative trend was the same: All ∆*cbrA hfq** mutants expressed *rhlAB* higher than ∆*cbrA crc** mutants but less than the wild-type. Each data bar shown with 95% confidence intervals obtained from 3 biological replicates.


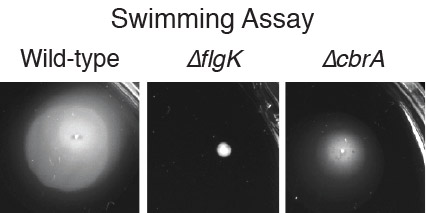


**Figure S3. Swimming assays conducted in soft agar (0.3 %) indicate that ∆*cbrA* despite being incapable of swarming continues to have functional flagella.** The wild-type (positive control) and ∆*cbrA* were both capable of swimming while ∆*flgK* (negative control) was not.


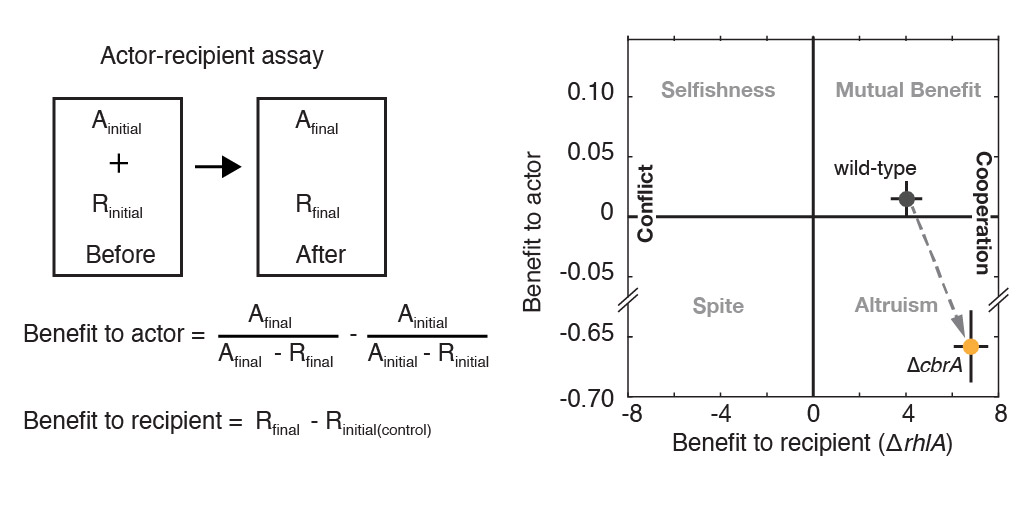


**Figure S4. The “actor-recipient” assay used to compare the social behaviors of *P. aeruginosa* strains.** The wild-type, which is capable of metabolic prudence, could help a ∆*rhlA* strain providing a positive benefit to this recipient strain without itself suffering a cost (non-negative benefit to actor). The ∆*cbrA* strain on the other hand helped the ∆*rhlA* recipient but at a high cost to its own fitness (negative benefit to actor). Therefore, by creating the ∆*cbrA* we engineered a synthetic altruist.


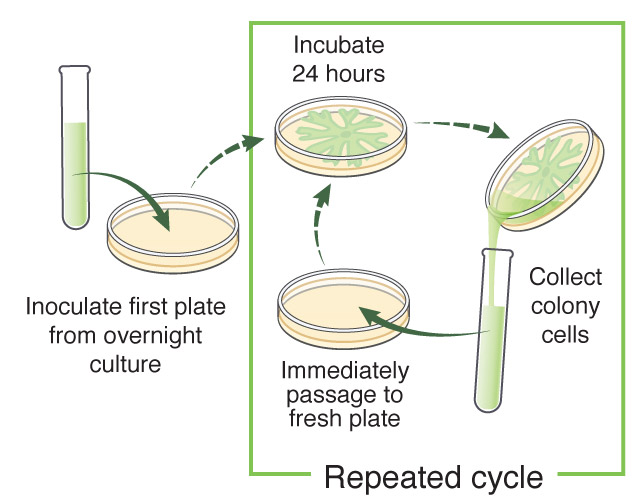


**Figure S5. Experimental evolution design (see methods).**


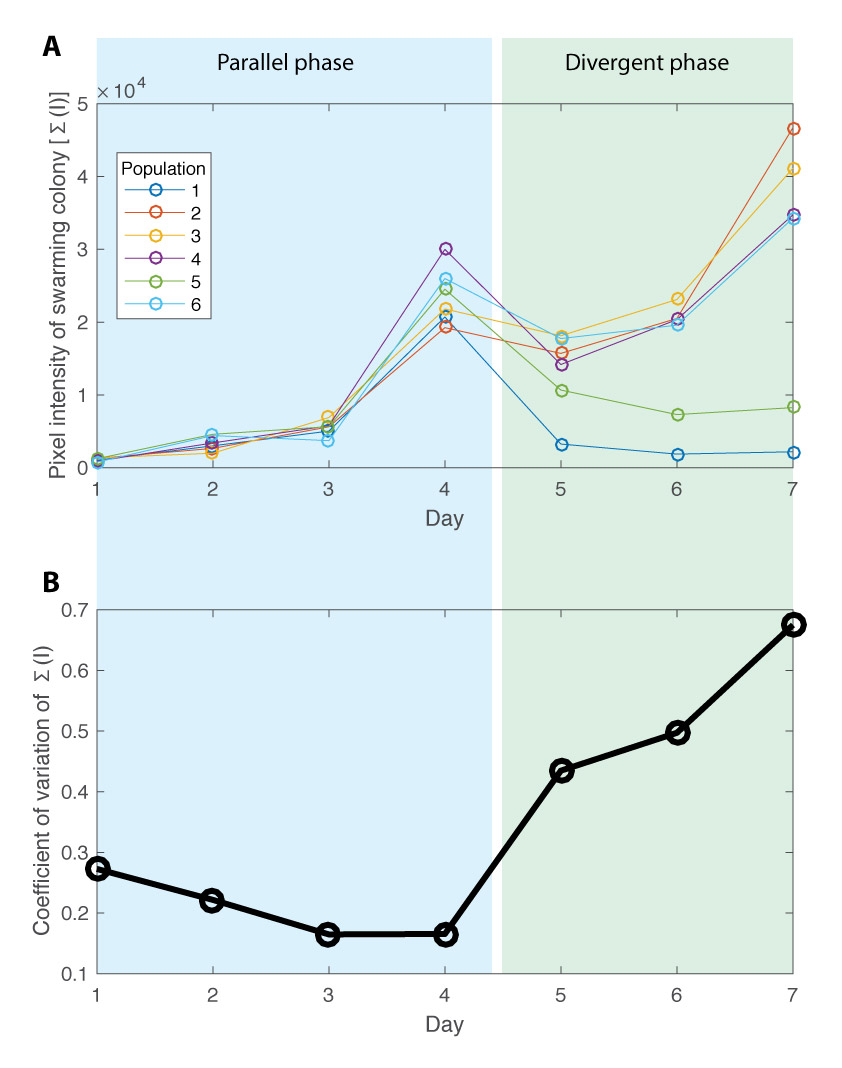


**Figure S6. Parallel evolution transitions to divergent evolution between days 4 and 5. A.** Image analysis of swarming colony pictures conducted in Matlab quantified the summed pixel intensities from each colony. The value of the summed intensity increased in every population until day 4 but started diverging after day 4. Specifically, populations 1 and 5 decreased drastically, while the others sustained or even expanded swarming. **B.** The transition at day 4 was even clearer by calculating the coefficient of variation from the six colonies. The variation decreased until day 4, supporting parallel evolution, and increased sharply at day 5 and upward as the six populations diverged in the divergent phase.

**
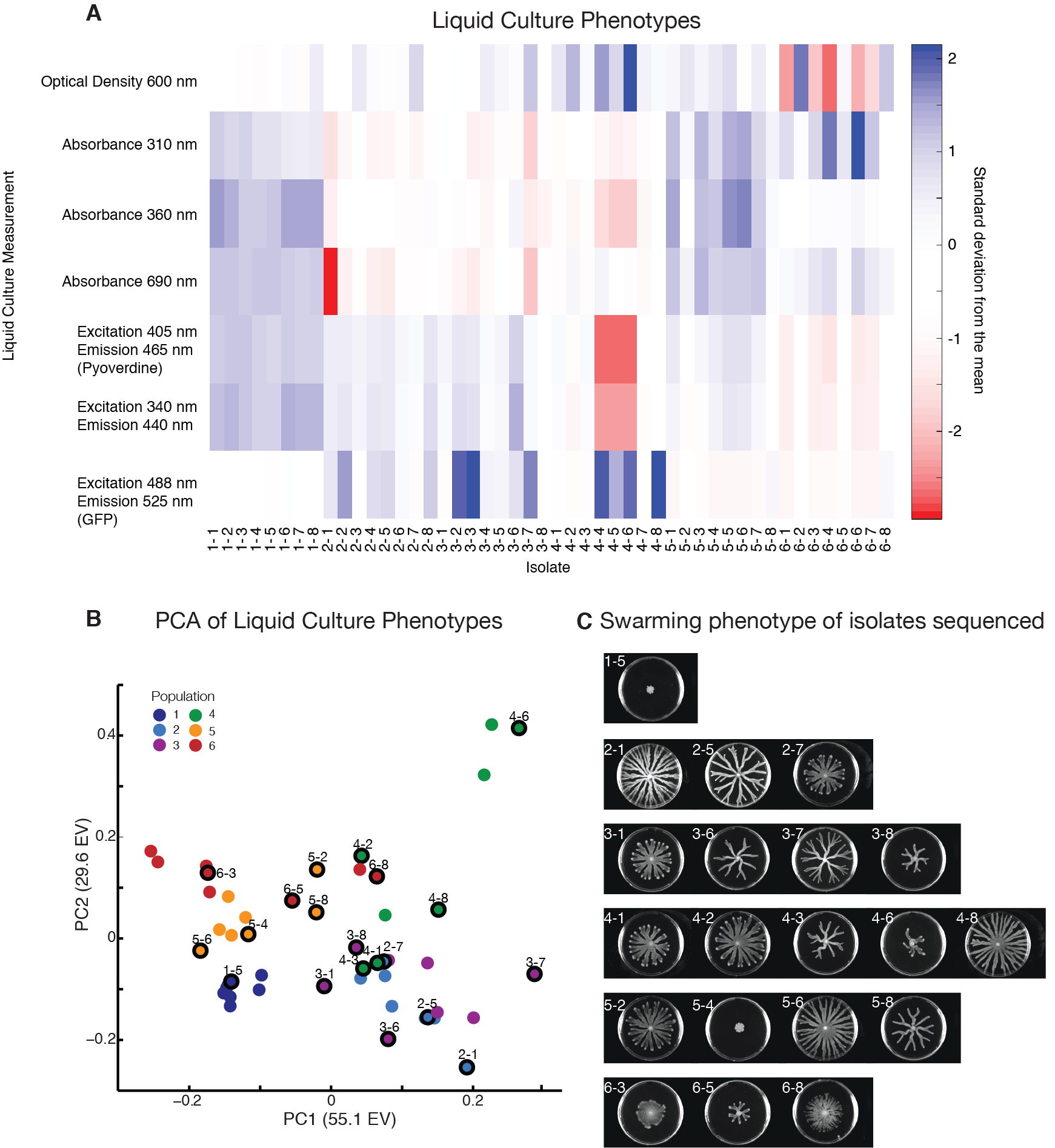
**

**Figure S7. Selection of 20 isolates down from the 48 isolates, eight collected from each of the six populations at day 7. A.** Normalized heat map of several spectrometric phenotypic measurements done in liquid culture after overnight growth at 37˚C in casamino acids media. Absorbance was measured at 600 nm, 690 nm, and 310 nm. Fluorescent measurements were made at an excitation of 488 nm of and an emission of 525 nm (GFP), an excitation of 405nm and an emission of 465 nm (Pyoverdine, pvd), and an excitation of 340 nm and an emission of 440 nm. **B.** Principal component analysis of the 48 isolates using the liquid culture phenotype information. The 20 isolates selected for sequencing aimed to sample the clonal diversity across all populations at day 7. **C.** Swarming colonies of the 20 selected clones, showing a wide range of swarming phenotypes including loss of swarming (clones 1-5 and 5-4). Clones 3-1 (a ∆*cbrA crc** mutant) and clone 2-1 (a ∆*cbrA hfq** mutant) were selected for metabolomics and social behavior analysis.**
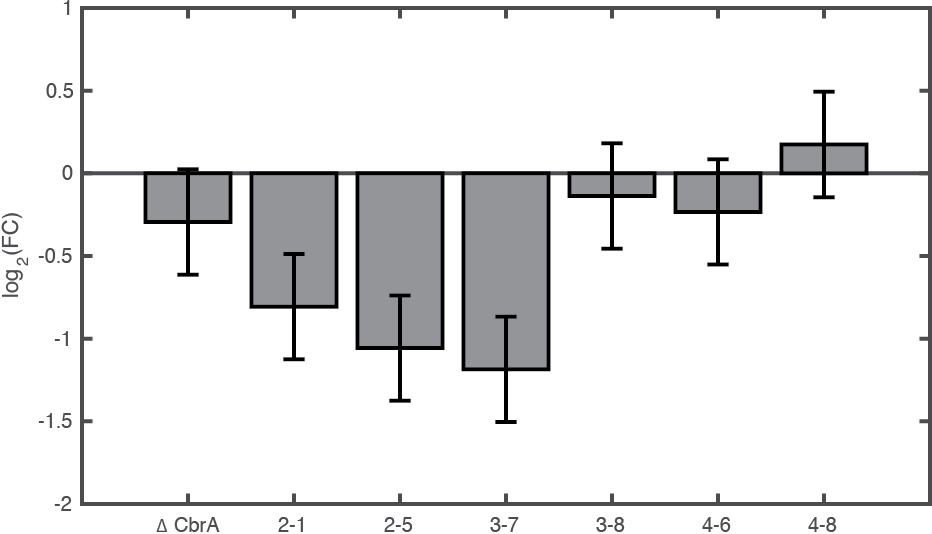
**

**Figure S8. Quantitative PCR to compare the *hfq* expression in all ∆*cbrA hfq** mutants isolated at day 7.** Fold change from wild-type of *hfq*. The three isolates with mutations upstream of *hfq* (2-1, 2-5 and 3-7) have lower levels of the *hfq* transcript while the isolates with mutations in the coding region (3-8, 4-6 and 4-8) have levels indistinguishable from the wild-type. Error bars represent 95% confidence intervals calculated from linear regression model (see methods).

**
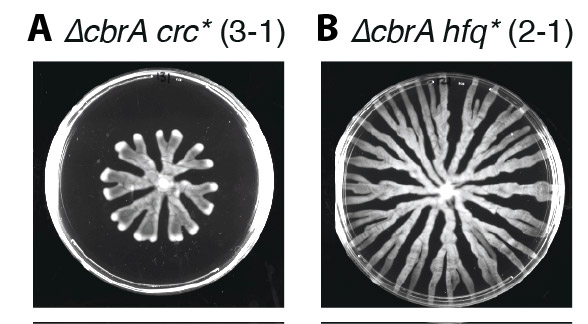
**

**Figure S9. Swarming colonies of isolate 3-1 (representative ∆*cbrA* *crc**) and isolate 2-1 (representative ∆*cbrA* *hfq**). Isolate 3-1 swarms less than the wild-type whereas 2-1 can swarm all the way to the edge.**


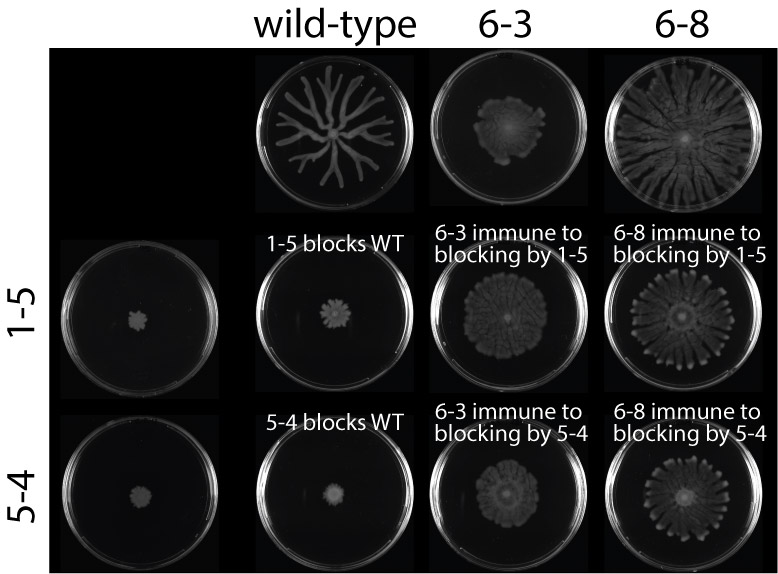


**Figure S10. Immotile isolates 1-5 and 5-4 block wild-type swarming, but are incapable of blocking hyperswarmer mutants 6-3 and 6-8.** The non-swarmer 1-5 and 5-4 have mutations in flagella-related loci (*fleR* and *fleQ*) and are immotile. These mutants could block the wild-type from swarming. Isolates 6-3 and 6-8, which have mutations in *fleN* known to create hyperswarmers, were immune to blocking.


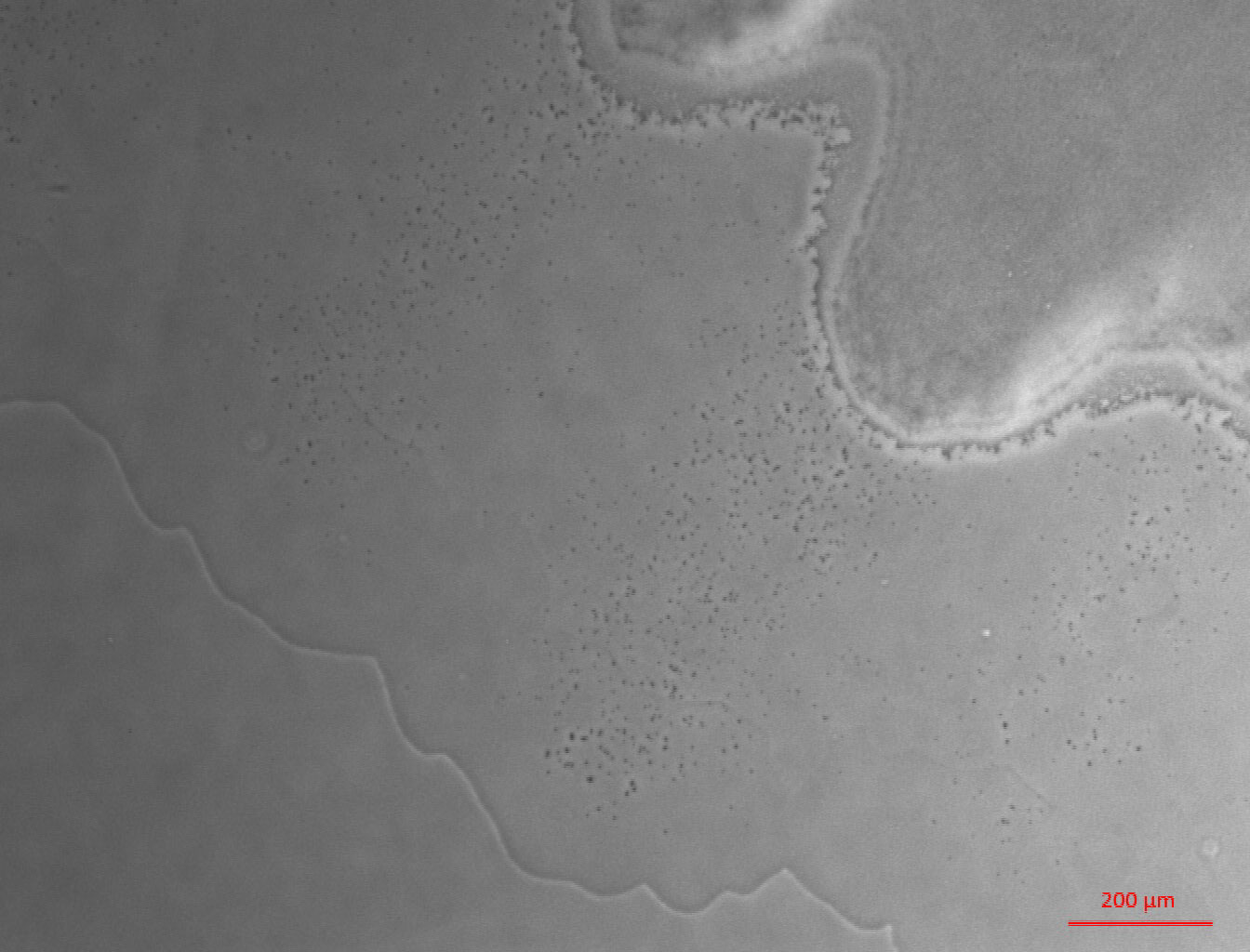


**Supporting video 1. Microscopy time-lapse of edge of swarming colony, showing the advancing of rhamnolipid biosurfactants spreading ahead of the colony.**


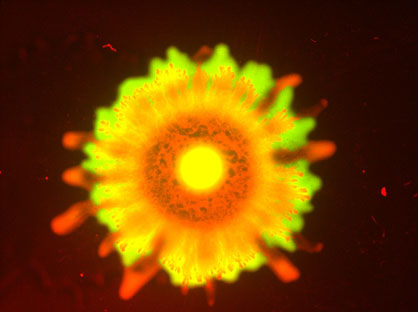


**Supporting video 2. The blocking effect of flagella-less bacteria.** *P. aeruginosa* lacking flagella (here, PA14 ∆*flgK* labeled with GFP) could prevent a swarming strain from swarming (here, wild-type PA14 labeled with DsRedExpress). The video illustrates that if the flagellated bacteria started out at a high enough ratio—here a 10:1 ratio—they could eventually pierce through the wall of non-flagellated bacteria.

**Table S1** Strains used in this study

| Strain | Description | Source |
| --- | --- | --- |
| Wild-type P*_rhlAB_-gfp* | The strain used as a background to delete *cbrA* locus | 1 |
| ∆*cbrA* P*_rhlAB_-gfp* | Ancestral strain used in all experimental evolution populations | This study |
| ∆*rhlA* P_A1/04/03_ *dsRedExpress* | Non-producer of rhamnolipids used for “actor-recipient” assays | 1 |
| ∆*rhlA* P*_rhlAB_-gfp* | Non-producer of rhamnolipids used to quantify the baseline value in “actor-recipient” assay | 2 |
| ∆*flgK* P_A1/04/03_ *gfp* | Flagella-less strain used for blocking experiments | This study |
| Wild-type P_A1/04/03_ *dsRedExpress* | Wild-type strain used for blocking experiments | 1 |
| FleNV178G P_A1/04/03_ *dsRedExpress* | Hyperswarmer strain used for blocking experiments | 3 |

**Sources:**

1. Xavier JB, Kim W, Foster KR (2011) A molecular mechanism that stabilizes cooperative secretions in Pseudomonas aeruginosa. Molecular microbiology.

2. van Ditmarsch D, Xavier J (2011) High-resolution time series of Pseudomonas aeruginosa gene expression and rhamnolipid secretion through growth curve synchronization. BMC microbiology 11: 140.

3. van Ditmarsch D, Boyle KE, Sakhtah H, Oyler JE, Nadell CD, et al. (2013) Convergent evolution of hyperswarming leads to impaired biofilm formation in pathogenic bacteria. Cell Rep 4: 697-708.

**Table S2** Mutations identified from whole genome sequencing and Breseq analysis of 20 isolates obtained from day 7 populations. The arrows in the Gene column indicate the direction of transcription for each gene. The arrow in the mutation column represent genetic changes; for example “G→A” means a change from guanine to adenine. Mutations highlighted in gray are trivial artifacts of the P*_rhlAB_*-*gfp* reporter construct (see **table S3**); Breseq calls these mutations in 7 isolates, and not in all isolates, because the reads map both to the native and to the inserted sequence and the coverage needed to call a variant is subject to the random mapping of reads to reference. These P*_rhlAB_*-*gfp* mutations were deemed artifacts as omitted from further analysis. Highlighted in yellow are the mutations called due to the excision of the 2,868 bp region in the *cbrA* locus used to make Δ*cbrA.* All 20 strains derive from Δ*cbrA* and therefore all harbor the deletion. Genomic sequences deposited in the Short Read Archive of the NCBI with accession number SRP103014.

| **Strain** | **Position** | **Mutation** | **Annotation** | **Gene** | **Description of mutation location** |
| --- | --- | --- | --- | --- | --- |
| **1-5** | 732,660 | G→A | intergenic (+114/‑435) | *tyrZ → / → PA14_08570* | tyrosyl‑tRNA synthetase 2/16S ribosomal RNA |
|  | 3,645,629 | (T)9→8 | intergenic (+60/‑198) | *PA14_40860 → / → PA14_40880* | putative sterol carrier protein/putative aminoglycoside phosphotransferase |
|  | 4,458,707 | Δ1 bp | coding (840/1422 nt) | *fleR ←* | two‑component response regulator |
|  | 6,275,314 | Δ2 bpcoding | (275‑276/780 nt) | *crc →* | catabolite repression control protein |
|  | 5,575,951 | Δ2,868 bp | coding (10‑2877/2952 nt) | *cbrA →* | two‑component sensor CbrA |
| **2-1** | 5,820,047 | C→T | intergenic (‑12/+94) | *hfq ← / ← miaA* | putative host factor‑I protein/delta 2‑isopentenylpyrophosphate transferase |
|  | 732,660 | G→A | intergenic (+114/‑435) | *tyrZ → / → PA14_08570* | tyrosyl‑tRNA synthetase 2/16S ribosomal RNA |
|  | 5,575,951 | Δ2,868 bp | coding (10‑2877/2952 nt) | *cbrA →* | two‑component sensor CbrA |
| **2-5** | 5,820,047 | C→T | intergenic (‑12/+94) | *hfq ← / ← miaA* | putative host factor‑I protein/delta 2‑isopentenylpyrophosphate transferase |
|  | 732,660 | G→A | intergenic (+114/‑435) | *tyrZ → / → PA14_08570* | tyrosyl‑tRNA synthetase 2/16S ribosomal RNA |
|  | 1,648,344 | A→G | intergenic (+136/‑288) | *dcd → / → rhlA* | trivial artifact of the P*_rhlAB_*-*gfp* reporter |
|  | 2,545,663 | T→C | I527I (ATT→ATC) | *PA14_29390 →* | conserved hypothetical protein |
|  | 5,575,951 | Δ2,868 bp | coding (10‑2877/2952 nt) | *cbrA →* | two‑component sensor CbrA |
| **2-7** | 6,275,182 | Δ1 bp | coding (143/780 nt) | *crc →* | catabolite repression control protein |
|  | 5,575,951 | Δ2,868 bp | coding (10‑2877/2952 nt) | *cbrA →* | two‑component sensor CbrA |
| **3-1** | 6,275,268 | A→T | K77* (AAA→TAA) | *crc →* | catabolite repression control protein |
|  | 5,575,951 | Δ2,868 bp | coding (10‑2877/2952 nt) | *cbrA →* | two‑component sensor CbrA |
| **3-6** | 6,275,593 | T→C | L185P (CTC→CCC) | *crc →* | catabolite repression control protein |
|  | 1,648,344 | A→G | intergenic (+136/‑288) | *dcd → / → rhlA* | trivial artifact of the P*_rhlAB_*-*gfp* reporter |
|  | 5,575,951 | Δ2,868 bp | coding (10‑2877/2952 nt) | *cbrA →* | two‑component sensor CbrA |
| **3-7** | 2,545,663 | T→C | I527I (ATT→ATC) | *PA14_29390 →* | conserved hypothetical protein |
|  | 5,820,094 | G→C | intergenic (‑59/+47) | *hfq ← / ← miaA* | putative host factor‑I protein/delta 2‑isopentenylpyrophosphate transferase |
|  | 5,575,951 | Δ2,868 bp | coding (10‑2877/2952 nt) | *cbrA →* | two‑component sensor CbrA |
| **3-8** | 5,819,890 | G→C | T49S (ACC→AGC) | *hfq ←* | putative host factor‑I protein |
|  | 1,648,588 | A→C | intergenic (+380/‑44) | *dcd → / → rhlA* | trivial artifact of the P*_rhlAB_*-*gfp* reporter |
|  | 3,645,629 | (T)9→8 | intergenic (+60/‑198) | *PA14_40860 → / → PA14_40880* | putative sterol carrier protein/putative aminoglycoside phosphotransferase |
|  | 5,575,951 | Δ2,868 bp | coding (10‑2877/2952 nt) | *cbrA →* | two‑component sensor CbrA |
| **4-1** | 6,275,443 | +A | coding (404/780 nt) | *crc →* | catabolite repression control protein |
|  | 2,545,633 | T→C | H517H (CAT→CAC) | *PA14_29390 →* | conserved hypothetical protein |
|  | 2,545,663 | T→C | I527I (ATT→ATC) | *PA14_29390 →* | conserved hypothetical protein |
|  | 5,575,951 | Δ2,868 bp | coding (10‑2877/2952 nt) | *cbrA →* | two‑component sensor CbrA |
| **4-2** | 6,275,529 | Δ60 bp | coding (490‑549/780 nt) | *crc →* | catabolite repression control protein |
|  | 1,648,344 | A→G | intergenic (+136/‑288) | *dcd → / → rhlA* | trivial artifact of the P*_rhlAB_*-*gfp* reporter |
|  | 5,575,951 | Δ2,868 bp | coding (10‑2877/2952 nt) | *cbrA →* | two‑component sensor CbrA |
| **4-3** | 6,275,604 | G→A | D189N (GAC→AAC) | *crc →* | catabolite repression control protein |
|  | 3,577,158 | T→C | M117V (ATG→GTG) | *polB ←* | DNA polymerase II |
|  | 4,659,831 | Δ1 bp | intergenic (+71/+31) | *PA14_52530 → / ← PA14_52540* | probable transcriptional regulator/tRNA‑Arg |
|  | 5,575,951 | Δ2,868 bp | coding (10‑2877/2952 nt) | *cbrA →* | two‑component sensor CbrA |
| **4-6** | 5,819,846 | G→C | P64A (CCG→GCG) | *hfq ←* | putative host factor‑I protein |
|  | 2,910,713 | C→A | intergenic (‑96/‑548) | *pvdS ← / → pvdG* | sigma factor PvdS/PvdG |
|  | 5,575,951 | Δ2,868 bp | coding (10‑2877/2952 nt) | *cbrA →* | two‑component sensor CbrA |
| **4-8** | 5,819,840 | G→A | R66C (CGT→TGT) | *hfq ←* | putative host factor‑I protein |
|  | 732,660 | G→A | intergenic (+114/‑435) | *tyrZ → / → PA14_08570* | tyrosyl‑tRNA synthetase 2/16S ribosomal RNA |
|  | 1,441,862 | (C)6→5 | intergenic (+32/+11) | *PA14_16820 → / ← PA14_16830* | putative efflux transmembrane protein/conserved hypothetical protein |
|  | 3,673,534 | T→C | intergenic (+83/+15) | *fabI → / ← ppiD* | NADH‑dependent enoyl‑ACP reductase/putative peptidyl‑prolyl cis‑trans isomerase D |
|  | 3,673,536 | T→C | intergenic (+85/+13) | *fabI → / ← ppiD* | NADH‑dependent enoyl‑ACP reductase/putative peptidyl‑prolyl cis‑trans isomerase D |
|  | 4,659,831 | Δ1 bp | intergenic (+71/+31) | *PA14_52530 → / ← PA14_52540* | probable transcriptional regulator/tRNA‑Arg |
|  | 5,575,951 | Δ2,868 bp | coding (10‑2877/2952 nt) | *cbrA →* | two‑component sensor CbrA |
| **5-2** | 6,275,479 | G→A | G147D (GGC→GAC) | *crc →* | catabolite repression control protein |
|  | 732,660 | G→A | intergenic (+114/‑435) | *tyrZ → / → PA14_08570* | tyrosyl‑tRNA synthetase 2/16S ribosomal RNA |
|  | 5,575,951 | Δ2,868 bp | coding (10‑2877/2952 nt) | *cbrA →* | two‑component sensor CbrA |
| **5-4** | 6,275,139 | C→T | Q34* (CAG→TAG) | *crc →* | catabolite repression control protein |
|  | 4,461,536 | A→C | V270G (GTC→GGC) | *fleQ ←* | transcriptional regulator FleQ |
|  | 1,648,588 | A→C | intergenic (+380/‑44) | *dcd → / → rhlA* | putative deoxycytidine triphosphate deaminase/rhamnosyltransferase chain A |
|  | 3,645,629 | (T)9→8 | intergenic (+60/‑198) | *PA14_40860 → / → PA14_40880* | putative sterol carrier protein/putative aminoglycoside phosphotransferase |
|  | 5,575,951 | Δ2,868 bp | coding (10‑2877/2952 nt) | *cbrA →* | two‑component sensor CbrA |
| **5-6** | 6,275,757 | Δ9 bp | coding (718‑726/780 nt) | *crc →* | catabolite repression control protein |
|  | 732,660 | G→A | intergenic (+114/‑435) | *tyrZ → / → PA14_08570* | tyrosyl‑tRNA synthetase 2/16S ribosomal RNA |
|  | 4,659,831 | Δ1 bp | intergenic (+71/+31) | *PA14_52530 → / ← PA14_52540* | probable transcriptional regulator/tRNA‑Arg |
|  | 5,575,951 | Δ2,868 bp | coding (10‑2877/2952 nt) | *cbrA →* | two‑component sensor CbrA |
| **5-8** | 6,275,757 | Δ9 bp | coding (718‑726/780 nt) | *crc →* | catabolite repression control protein |
|  | 1,648,344 | A→G | intergenic (+136/‑288) | *dcd → / → rhlA* | trivial artifact of the P*_rhlAB_*-*gfp* reporter |
|  | 5,575,951 | Δ2,868 bp | coding (10‑2877/2952 nt) | *cbrA →* | two‑component sensor CbrA |
| **6-3** | 6,275,529 | Δ60 bp | coding (490‑549/780 nt) | *crc →* | catabolite repression control protein |
|  | 4,060,176 | G→A | S28L (TCG→TTG) | *fleN ←* | flagellar synthesis regulator FleN |
|  | 1,648,588 | A→C | intergenic (+380/‑44) | *dcd → / → rhlA* | trivial artifact of the P*_rhlAB_*-*gfp* reporter |
|  | 1,648,631 | G→A | intergenic (+423/‑1) | *dcd → / → rhlA* | trivial artifact of the P*_rhlAB_*-*gfp* reporter |
|  | 5,575,951 | Δ2,868 bp | coding (10‑2877/2952 nt) | *cbrA →* | two‑component sensor CbrA |
| **6-5** | 6,275,529 | Δ60 bp | coding (490‑549/780 nt) | *crc →* | catabolite repression control protein |
|  | 5,575,951 | Δ2,868 bp | coding (10‑2877/2952 nt) | *cbrA →* | two‑component sensor CbrA |
| **6-8** | 6,275,007 | T→C | intergenic (‑48/‑33) | *pyrE ← / → crc* | orotate phosphoribosyltransferase/catabolite repression control protein |
|  | 4,059,726 | A→C | V178G (GTC→GGC) | *fleN ←* | flagellar synthesis regulator FleN |
|  | 5,575,951 | Δ2,868 bp | coding (10‑2877/2952 nt) | *cbrA →* | two‑component sensor CbrA |

**Table S3.** Sequence comparison of the 628 bp fragment of the P. aeruginosa PAO1 promoter of *rhlAB* included in plasmid pYL122 used to insert the P*_rhlAB_*-*gfp* reporter fusion into PA14 and the matching sequence from the promoter of *rhlAB* in the genome of PA14. The alignment shows four mismatches, which would be occasionally called as mutations by Breseq. Those mutations, marker in grey in table S2, and were excluded from downstream analysis. In Fig S1 we show that despite the four mismatches the transcription of *gfp* matched that of *rhlA*, confirming that this is a suitable reporter of *rhlA* transcription in PA14.

Multiple sequence alignment

2 Sequences Aligned Alignment Score = 0

Gaps Inserted = 0 Conserved Identities = 624

pYL122 1 GATCTACGCCAA**T**GAAGGCGTGGCGCAGATGCTCTTCCTGCAATCCGACGAGGCCTGCGA 60

PA14 1 GATCTACGCCAA**C**GAAGGCGTGGCGCAGATGCTCTTCCTGCAATCCGACGAGGCCTGCGA 60

************ ***********************************************

pYL122 61 AGTGTCCTATAAGGACCGTGGCGGCAAATACCAGGGCCAGCGCGGCGTGACCCTGCCAAA 120

PA14 61 AGTGTCCTATAAGGACCGTGGCGGCAAATACCAGGGCCAGCGCGGCGTGACCCTGCCAAA 120

************************************************************

pYL122 121 AGCCTGACGCCAGAGCGTTTCGACACCGGAAACCGGGCCTGGCGCCCGGTTTTTTCATGC 180

PA14 121 AGCCTGACGCCAGAGCGTTTCGACACCGGAAACCGGGCCTGGCGCCCGGTTTTTTCATGC 180

************************************************************

pYL122 181 CTTTTCCGCCAACCCCTCGCTGTTCCCCGCCGGCCGCTCTGGCACGCCTTATCGCGGGCG 240

PA14 181 CTTTTCCGCCAACCCCTCGCTGTTCCCCGCCGGCCGCTCTGGCACGCCTTATCGCGGGCG 240

************************************************************

pYL122 241 GGCAGGGGCTTATGCGCAGGCG**G**CCGCCCGTCCTGTGAAATCTGGCAGTTACCGTTAGCT 300

PA14 241 GGCAGGGGCTTATGCGCAGGCG**A**CCGCCCGTCCTGTGAAATCTGGCAGTTACCGTTAGCT 300

********************** *************************************

pYL122 301 TTCGAATTGGCTAAAAAGTGTTCATCGGCTACGCGTGAACACGGACGCCAATCGTTTGCG 360

PA14 301 TTCGAATTGGCTAAAAAGTGTTCATCGGCTACGCGTGAACACGGACGCCAATCGTTTGCG 360

************************************************************

pYL122 361 CAGGCCGATCTGCAAGACCCACACAAGCCCCTCGCCTGAAGGGGTACGCATCCGCCGTGG 420

PA14 361 CAGGCCGATCTGCAAGACCCACACAAGCCCCTCGCCTGAAGGGGTACGCATCCGCCGTGG 420

************************************************************

pYL122 421 CTGGTCCGCGCGGATGGCCGCTGAGTTACTTGTCTGCCGTTCGAACAATAAGAACGAACT 480

PA14 421 CTGGTCCGCGCGGATGGCCGCTGAGTTACTTGTCTGCCGTTCGAACAATAAGAACGAACT 480

************************************************************

pYL122 481 CTACGTAATGCCGGGATACCCGTGGC**C**GCGATAGCTGTTTGCCTGTTCGAAAATTTTTGG 540

PA14 481 CTACGTAATGCCGGGATACCCGTGGC**A**GCGATAGCTGTTTGCCTGTTCGAAAATTTTTGG 540

************************** *********************************

pYL122 541 GAGGTGTGA**A**ATGCGGCGCGAAAGTCTGTTGGTATCGGTTTGCAAGGGCCTGCGGGTACA 600

PA14 541 GAGGTGTGA**G**ATGCGGCGCGAAAGTCTGTTGGTATCGGTTTGCAAGGGCCTGCGGGTACA 600

********* **************************************************

pYL122 601 TGTCGAGCGCGTTGGGCAGGATCCCGGG 628

PA14 601 TGTCGAGCGCGTTGGGCAGGATCCCGGG 628

****************************
